# Mutations in the Heme Exporter *FLVCR1* Cause Sensory Neurodegeneration with Loss of Pain Perception

**DOI:** 10.1371/journal.pgen.1006461

**Published:** 2016-12-06

**Authors:** Deborah Chiabrando, Marco Castori, Maja di Rocco, Martin Ungelenk, Sebastian Gießelmann, Matteo Di Capua, Annalisa Madeo, Paola Grammatico, Sophie Bartsch, Christian A. Hübner, Fiorella Altruda, Lorenzo Silengo, Emanuela Tolosano, Ingo Kurth

**Affiliations:** 1 Department of Molecular Biotechnology and Health Sciences, Molecular Biotechnology Center, University of Torino, Torino, Italy; 2 Unit of Medical Genetics, Department of Molecular Medicine, Sapienza University, San Camillo-Forlanini Hospital, Rome, Italy; 3 Unit of Rare Diseases, Department of Pediatrics, Gaslini Institute, Genoa, Italy; 4 Institute of Human Genetics, Jena University Hospital, Friedrich-Schiller-University Jena, Jena, Germany; 5 Unit of Neurophysiopathology, Department of Neuroscience, Bambino Gesù Children's Hospital, Rome, Italy; 6 Institute of Human Genetics, Uniklinik RWTH Aachen, Aachen, Germany; Columbia University Medical Center, UNITED STATES

## Abstract

Pain is necessary to alert us to actual or potential tissue damage. Specialized nerve cells in the body periphery, so called nociceptors, are fundamental to mediate pain perception and humans without pain perception are at permanent risk for injuries, burns and mutilations. Pain insensitivity can be caused by sensory neurodegeneration which is a hallmark of hereditary sensory and autonomic neuropathies (HSANs). Although mutations in several genes were previously associated with sensory neurodegeneration, the etiology of many cases remains unknown. Using next generation sequencing in patients with congenital loss of pain perception, we here identify bi-allelic mutations in the *FLVCR1* (Feline Leukemia Virus subgroup C Receptor 1) gene, which encodes a broadly expressed heme exporter. Different FLVCR1 isoforms control the size of the cytosolic heme pool required to sustain metabolic activity of different cell types. Mutations in *FLVCR1* have previously been linked to vision impairment and posterior column ataxia in humans, but not to HSAN. Using fibroblasts and lymphoblastoid cell lines from patients with sensory neurodegeneration, we here show that the FLVCR1-mutations reduce heme export activity, enhance oxidative stress and increase sensitivity to programmed cell death. Our data link heme metabolism to sensory neuron maintenance and suggest that intracellular heme overload causes early-onset degeneration of pain-sensing neurons in humans.

## Author Summary

Hereditary Sensory and Autonomic Neuropathy (HSAN) is a genetic disorder mainly characterized by the impairment of sensory neurons, which transmit information about sensations such as pain, temperature and touch. Therefore, unintentional self-injury, leading to ulcers and eventually amputations are common in affected individuals. Although mutations in several genes were previously associated with sensory neurodegeneration and pain insensitivity, the etiology of many cases remains unknown. We here identify mutations in the heme exporter protein FLVCR1 in patients with congenital inability to experience pain. We showed that FLVCR1 mutations results in reduced heme export activity, enhanced oxidative stress and increased sensitivity to programmed cell death. These data assign a surprising role for heme to sensory neuron maintenance.

## Introduction

Neurodegenerative disorders affecting peripheral sensory neurons lead to loss of pain perception as disease hallmark. The absence of protective behaviors towards noxious stimuli causes unintentional self-injuries and chronic ulcerations. Soft tissue infections and osteomyelitis, often requiring amputations, are common and complicate this disorder [1, 2]. Autonomic dysfunction and motor deficits may be additional features of sensory and autonomic neuropathies (HSANs). Prominent loss of large and small myelinated fibers distinguishes sensory neuropathies from clinically similarly presenting channelopathy-associated pain insensitivity (CIP)[3].

Proteins which are involved in sensory neurodegeneration affect distinct molecular pathways: sphingolipid-metabolism, membrane-shaping of organelles, regulation of ion channels, endoplasmic reticulum turnover and axonal trafficking[1, 4–8]. However, the molecular mechanisms underlying sensory neurodegeneration are still incompletely understood and disease-causing mutations remain to be identified in a substantial number of patients.

Rapid progress in next-generation sequencing (NGS) technology has transformed the field of medical genomics leading to the identification of novel disease-genes[9, 10]. In this study, next generation sequencing was performed in patients with HSAN but without mutations in the known genes associated with the disorder. Causative mutations were found in *FLVCR1* (Feline Leukemia Virus subgroup C Receptor 1), a gene that has previously been associated to Posterior Column Ataxia and Retinitis Pigmentosa (PCARP)[11–16].

FLVCR1 is an ubiquitously expressed heme exporter[17, 18], member of the Major Facilitator Superfamily (MFS) transporters[19]. Two different isoforms have been described. FLVCR1a resides in the plasma membrane and is responsible for heme detoxification in several cell types, such as erythroid progenitors, endothelial cells, hepatocytes, lymphocytes and intestinal cells[18, 20–25]. FLVCR1b is located on mitochondria and is involved in the transport of newly synthesized heme from mitochondria to the cytosol[18]. The expression of FLVCR1a and FLVCR1b is needed to control the size of the cytoplasmic free-heme pool, which is essential for proper metabolic functions[21, 23]. Heme is an essential co-factor involved in multiple biological processes: oxygen transport and storage, electron transfer, drug and steroid metabolism, signal transduction and microRNA processing[26]. However, excess free-heme is highly toxic due to its ability to promote oxidative stress, proteasome inhibition and mitochondrial dysfunction, that ultimately lead to cell death[26–28]. For this reason, the intracellular free-heme pool is finely regulated at multiple levels[26] and FLVCR1a-mediated heme export contributes to this process[21, 22, 26]. Our data show that primary fibroblasts and lymphoblastoid cell lines from patients with sensory neurodegeneration have reduced heme export activity due to FLVCR1-mutations. This results in enhanced oxidative stress and increased sensitivity to programmed cell death. These data add heme metabolism to the molecular pathways implicated in sensory neuron maintenance and pain processing.

## Results

### Mutations in the *FLVCR1* gene cause sensory neurodegeneration with loss of pain perception in humans

**Fig 1:**
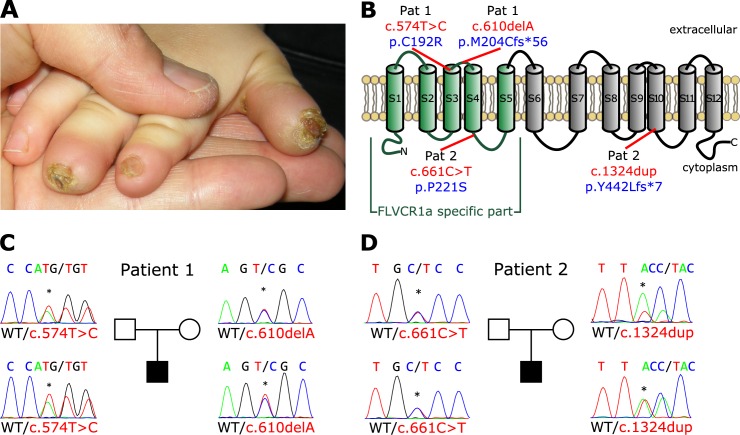
Mutations in the *FLVCR1* gene cause Sensory Neuropathy. (**A**) Non-healing wound lesions with nail dystrophy in patient 2 as clinical feature of sensory neuropathy caused by *FLVCR1* mutations. (**B**) Structure of the FLVCR1a protein (plasma membrane isoform). FLVCR1a-specific part is depicted in green. Mutations are indicated in the scheme, electropherograms with mutations and pedigrees are given for patient 1 (**C**) and patient 2 (**D**).

We studied trios with an affected child and healthy parents with the diagnosis of hereditary sensory and autonomic neuropathy (HSAN) (Fig 1 and Table 1). In an Italian boy with early-onset pain insensitivity (patient 1) and non-consanguineous parents whole-exome sequencing identified 559 variants with low prevalence (<0,01%) in dbSNP, the 1000-Genomes project, the Exome Variant Server or the ExAC browser. Variants were prioritized for *de novo* mutations and compound-heterozygous / homozygous variants (S1 Table). Amongst these variants compound-heterozygosity for mutations in *FLVCR1* (NM_014053.3) was detected (Fig 1B and 1C). The heterozygous mutation c.574T>C; p.(Cys192Arg) has been previously reported as homozygous mutation in autosomal-recessive posterior column ataxia with retinitis pigmentosa (PCARP)[14]. The respective cytosine is located in the third transmembrane domain of the FLVCR1 protein (Fig 1B) and is evolutionarily conserved among species. The mother of patient 1 is heterozygous carrier of the c.574T>C mutation. The second mutation was inherited from the father and is a heterozygous frameshift-mutation c.610del; p.(Met204Cysfs*56). The deletion results in a premature stop codon, hence suggesting complete loss of protein function. Both mutations were considered as likely pathogenic accordingly to *in-silico* prediction programs (S2 Table). To forecast gene-specific pathogenicity of the variants in the context of the *FLVCR1* gene, a Mutation Significance Cutoff (MSC) 23.8 for CADD, 0.697 for PolyPhen2 and 0.04 for SIFT was applied based on calculations by the MSC server for HGMD and ClinVar variants[29]. If applicable, all tools predicted a deleterious impact of the specific mutations (S2 Table).

**Table 1:** 

Feature	Patient 1	Patient 2
Age at examination	33 months	41 months
Sex	Male	Male
Consanguinity	-	-
Compound heterozygous *FLVCR1* mutations	c.574T>C; p.(Cys192Arg),	c.661C>T; p.(Pro221Ser),
	c.610del; p.(Met204Cysfs*56)	c.1324dup; p.(Tyr442Leufs*7)
Sensory neuropathy	+	+
Finger mutilations	+	+
Slow-healing ulcers/wounds	+	+
Osteomyelitis	-	-
Autonomic features	Autonomic “crises”, tearing dysfunction	-
Developmental delay	+ (severe)	+ (mild/moderate)
Ataxic gait	NE	+
Retinal involvement	+/-	+/-
Hepatic involvement	+	-
Bone marrow involvement	Hyporegenerative anemia	-
Hyperintensity of the posterior columns	NE	+
Syringomyelia	-	+
Brain atrophy	+	-
White matter anomalies	+	-

*Patient 1* was the only child of non-consanguineous parents and at 23 months of age presented with severe psychomotor delay, no language acquisition, absent reaction to painful stimuli, ulcero-mutilations of mouth and hands, joint hypermobility and scoliosis. Sensory nerve conduction was not evocable. The child had frequent crises resembling “dysautonomic crises”, characterized by agitation and tachycardia, spontaneously resolving after vomiting or stool emission. He had chronic macrocytic anemia, with normal ferritin, syderemia, transferrin, vitamin B12 and folic acid levels; bone marrow examination showed hyporegenerative anemia. At the age of 33 months he developed seizures. In the later disease course, optic atrophy and retinal degeneration were also noticed (Table 1). The clinical presentation thus showed a predominant pain insensitivity phenotype with clinical symptoms partially overlapping with PCARP, suggesting that distinct *FLVCR1* mutations may result in different clinical outcomes.

To substantiate the role of FLVCR1 in sensory neuropathies additional index cases and trios were subjected to next-generation sequencing using a gene panel consisting of 70 genes implicated in HSAN and similar syndromes, including *FLVCR1*. Amongst 165 index cases, we identified a second case with early-onset sensory neuropathy (patient 2) and compound heterozygous mutations c.661C>T; p.(Pro221Ser) and c.1324dup; p.(Tyr442Leufs*7) in *FLVCR1* (Fig 1B and 1D). The non-consanguineous family of European origin (mother, father, index patient) was further subjected to trio exome-sequencing to evaluate mutations in other than the initially tested genes. No other candidate gene which was likely to account for the clinical phenotype was identified by this approach (S1 Table).

Pro221 is an evolutionarily highly conserved amino acid residue of FLVCR1 and its mutation to serine is likely to interfere with FLVCR1 function. This missense exchange is extremely rare and accordingly to the Exome Aggregation Consortium (ExAC) has been observed in 1 of 120,990 alleles. Pro221Ser is also predicted as pathogenic (S2 Table). Again, the second FLVCR1 allele harbors a loss-of-function frameshift mutation, which has not been described until now.

Patient 2 had mild developmental delay and started to walk with support at age of 24 months with a broad base gait. Speech was also delayed with first words at 22 months. At 12 months of age a non-healing wound at the tip of the right thumb with nail dystrophy was noticed. Despite local medication necrosis and acroosteolysis occurred. Similar lesions appeared on the 2nd and 3rd right fingers and left thumb with loss of nails and resorption of the underlying soft tissues. Wounds were slow healing, resulting in atrophic scarring. At examination, the patient presented hyperactive and clumsy, and showed marked reduction to painful stimuli. Affected fingers had stubby ends and nail dystrophy (Fig 1A). He had a positive histamine axonal flare response, and electroneurography showed a severe sensory neuropathy. Electroencephalogram and brain MRI gave normal results, while total spine MRI showed syrinx from T5 to T10, minor irregularities of the cervical central ependymal canal, and mild hyperintensity of the posterior columns (S1 Fig). An electroretinogram, performed at 3 years, showed reduced a-wave amplitude suggestive for initial retinal epithelium degeneration. Together, these data link *FLVCR1* mutations to early-onset complicated sensory neuropathy.

### *FLVCR1* mutations impair plasma membrane heme export in patient-derived cells

**Fig 2:**
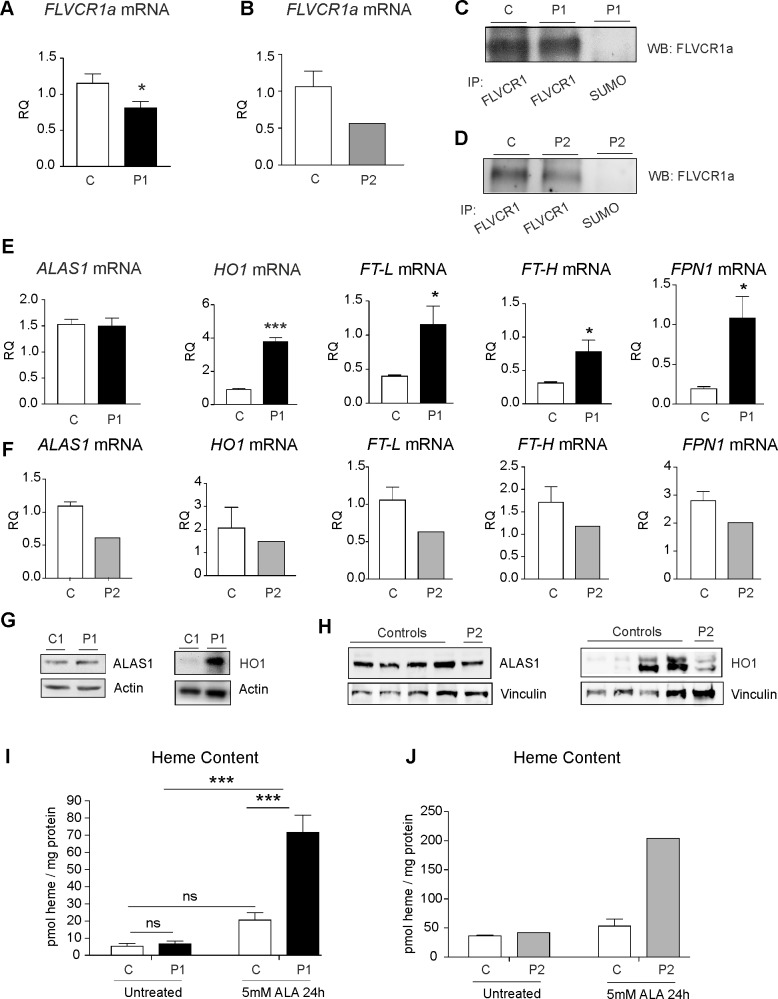
FLVCR1 mutations impair heme export in patient-derived cells. (**A**) qRT-PCR analysis of *FLVCR1a* mRNA in patient 1 compared to control fibroblasts (black). Values represent mean ± SEM. n = 6. * = P<0.05. (**B**) qRT-PCR analysis of *FLVCR1a* mRNA in patient 2 compared to control LCLs (grey). Values represent mean *FLVCR1a* mRNA levels in patient 2 compared to the mean *FLVCR1a* mRNA levels of 4 different control LCLs. (**C**) Immunoprecipitation and western blotting of FLVCR1a in patient 1 compared to control fibroblasts. A representative blot is shown. The antibody against SUMO was used as control. (**D**) Immunoprecipitation and western blotting of FLVCR1a in patient 2 compared to control LCLs. A representative blot is shown. The antibody against SUMO was used as control. (**E**) qRT-PCR analysis of *ALAS1*, *HO1*, *FT-L*, *FT-H and FPN1 mRNA* in patient compared to control fibroblasts. Values represent mean ± SEM. n = 6. * = P<0.05; *** = P<0.001. (**F**) qRT-PCR analysis of *ALAS1*, *HO1*, *FT-L*, *FT-H and FPN1 mRNA* in patient compared to control LCLs. Values represent mean mRNA levels in patient 2 compared to the mean mRNA levels of 4 different control LCLs. (**G**) Western blot analysis of HO1 and ALAS1 protein in patient 1 compared to control fibroblasts. A representative blot is shown. (**H**) Western blot analysis of ALAS1 and HO1 protein levels in patient 2 compared to 4 different control LCLs. A representative blot is shown. (**I**) Measurement of heme content in patient 1 compared to control fibroblasts. Values represent mean ± SEM. n = 6. Two-way ANOVA. *** = P<0.001. (**J**) Measurement of heme content in patient 1 compared to control LCLs. Values represent heme content of patient 2 LCLs compared to the mean heme content of 4 different control LCLs. P1 = patient 1, P2 = patient 2, C = control.

To investigate the functional consequences of *FLVCR1* mutations, primary fibroblasts were derived from patient 1 whereas lymphoblastoid cell lines (LCLs) were generated from patient 2. qRT-PCR analyses showed that *FLVCR1* mutations result in a specific decrease of *FLVCR1a* transcript whereas *FLVCR1b* mRNA levels were unaffected in both patients (Fig 2A and 2B and S2 Fig). Interestingly, immunoprecipitation and western blotting indicated that FLVCR1a is still expressed in patient fibroblasts and LCLs (Fig 2C and 2D). These data indicate that the mutations did not completely abolish FLVCR1a expression. Therefore, we hypothesized that the mutations could interfere with the heme export ability of FLVCR1, thus resulting in intracellular heme accumulation. To address this issue, we analyzed the expression of proteins involved in heme and iron metabolism. The expression of ALAS1 (δ-aminolevulinc acid synthase 1), the rate-limiting enzyme in the heme biosynthetic pathway, was similar in patient and control fibroblasts (Fig 2E and 2G). However, increased expression of the heme degrading enzyme HO1 (Heme oxygenase 1) was observed in patient compared to control fibroblasts (Fig 2E and 2G). HO1 catalyzes the degradation of heme into biliverdin, carbon monoxide and iron[30]. Consistent with the induction of HO1 in patient fibroblasts, increased mRNA levels of the iron exporter *FPN* (Ferroportin) and the iron-storage proteins *FT-L* (Ferritin L) and *FT-H* (Ferritin H) were observed in patient compared to control fibroblasts (Fig 2E). Contrary, patient LCLs were characterized by decreased ALAS1 and similar HO1 expression levels compared to healthy donors (Fig 2F, 2H and S3 Fig). Consistent with reduced ALAS1 expression, we observed a slight decrease of *FPN*, *FT-L* and *FT-H* mRNA levels in patient compared to control LCLs (Fig 2F).

To definitively demonstrate that FLVCR1 mutations impair plasma membrane export, the amount of intracellular heme concentration was determined. Heme content was comparable between patient and control fibroblasts (Fig 2I) and LCLs (Fig 2J) under resting conditions, likely due to the induction of HO1 in patient fibroblasts and the reduction of ALAS1 in patient LCLs (Fig 2E–2H). However, following the stimulation of heme synthesis with ALA (δ-aminolevulinic acid), heme accumulation was observed in patient compared to control fibroblasts (Fig 2I). Moreover, heme content was higher in patient LCLs compared to the mean heme content of 4 different healthy donors LCLs (Fig 2J). Taken together these data indicate that FLVCR1 mutations impair plasma membrane heme export resulting in a transient accumulation of heme that activates cell-specific detoxifying mechanisms: excess-heme is rapidly catabolized by HO1 in patient-derived fibroblasts while heme downregulates its own synthesis in patient-derived LCLs.

### *FLVCR1* mutations induce oxidative stress and enhance the sensitivity to programmed cell death in patient-derived cells

**Fig 3:**
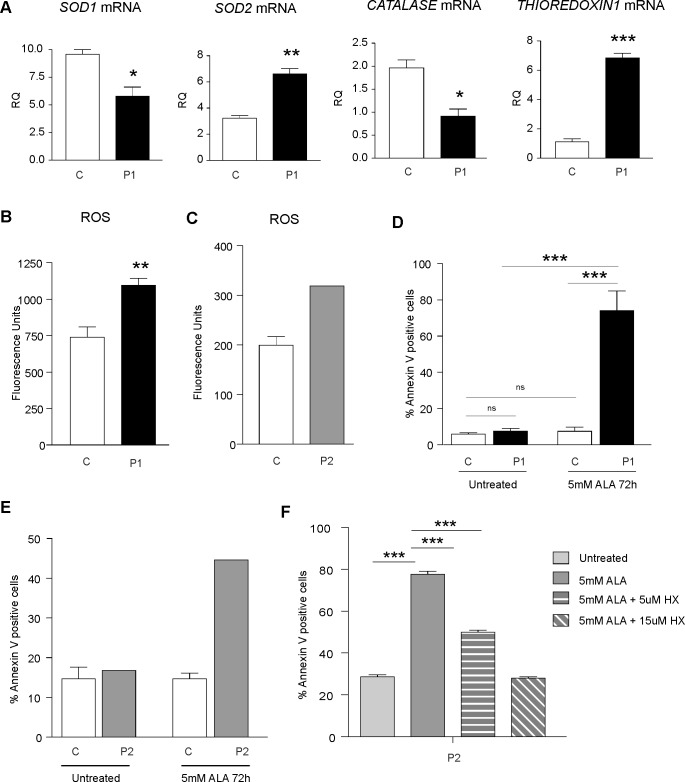
FLVCR1 mutations induce oxidative stress and increase the sensitivity to programmed cell death in patient-derived cells. (**A**) qRT-PCR analysis of *SOD1*, *SOD2*, *CATALASE* and *THIOREDOXIN1* mRNAs in patient 1 compared to control fibroblasts. Values represent mean ± SEM. n = 3. * = P<0.05; ** = P<0.005; *** = P<0.001. (**B**) Measurement of ROS levels in patient 1 compared to control fibroblasts. Values represent mean ± SEM. n = 6. ** = P<0.005. (**C**) Measurement of ROS levels in patient 2 compared to control LCLs. Values represent ROS levels of patient 2 LCLs compared to the mean heme content of 4 different control LCLs. (**D**) Percentage of Annexin V-positive cells in patient 1 compared to control fibroblasts, under basal conditions and following the stimulation with 5mM ALA for 72 hours. Values represent mean ± SEM. n = 6. Two-way ANOVA. *** = P<0.001. (**E**) Annexin V-positive cells in patient 2 compared to control LCLs under basal conditions and following the stimulation with 5mM ALA for 72 hours. Values represent the percentage of Annexin V-positive cells of patient 2 LCLs compared to the mean percentage of Annexin V-positive cells of 4 different control LCLs. (**F**) Annexin V-positive cells in patient LCLs grown in starved medium, treated with 5mM ALA and with or without Hemopexin (HX) for 24 hours. Values represent mean ± SEM. n = 6. Two-way ANOVA. *** = P<0.001. P1 = patient 1, P2 = patient 2, C = control.

Excess free-heme is highly toxic due to its ability to promote oxidative stress and lipid peroxidation, leading to membrane injury and, ultimately, apoptosis[26]. To evaluate whether *FLVCR1* mutations influence cellular oxidative status, the expression of genes involved in the antioxidant response was analyzed. Patient fibroblasts were characterized by decreased superoxide dismutase 1 (*SOD1*) and catalase mRNA levels, and increased superoxide dismutase 2 (*SOD2*) and thioredoxin transcripts (Fig 3A). Increased reactive oxygen species (ROS) were observed in patient compared to control fibroblasts (Fig 3B). Moreover, ROS levels were higher in patient LCLs compared to the mean ROS levels of 4 different healthy donors LCLs (Fig 3C).

In order to investigate whether loss of FLVCR1a affects cell viability, the survival of cells was evaluated by Annexin V staining. The percentage of Annexin V-positive cells was comparable between patient and control fibroblasts under resting conditions (Fig 3D). However, following the stimulation with ALA, increased percentage of Annexin V-positive cells was detected in patient compared to control fibroblasts (Fig 3D). In addition, following ALA treatment, the percentage of Annexin V-positive cells was higher in patient LCLs compared to the mean of 4 different healthy donors LCLs (Fig 3E). The same result was observed following the stimulation of patient and controls LCLs with H_2_O_2_ (S4 Fig). In order to demonstrate that increased apoptosis in patient cells treated with ALA was due to heme overload, we performed rescue experiments by reducing intracellular heme loading. It has been previously reported that Hemopexin (HX) facilitates heme export through FLVCR1[31, 32]. Thus, we added HX to the medium. HX treatment ameliorate cell survival in patient LCLs treated with ALA in a dose dependent manner (Fig 3F), thus suggesting that heme overload is responsible for the observed phenotype.

Taken together these data indicate that the impairment of FLVCR1-mediated heme export triggers oxidative stress and increases the susceptibility to programmed cell death in patient-derived cells.

### FLVCR1 is important for the survival of neuroblastoma cells

The identification of FLVCR1 mutations in patients with loss of pain perception, as well as PCARP, indicate that FLVCR1 plays a critical role in the nervous system. Previous studies confirmed the expression of FLVCR1 in the mouse brain, retina and spinal cord[14]. We analyzed *FLVCR1a* mRNA levels in different mouse tissues. *FLVCR1a* mRNA levels are higher in the brain compared to other tissues characterized by elevated production of hemoproteins (S5 Fig). This data suggests that FLVCR1a mediated heme export is relevant for neurons.

**Fig 4:**
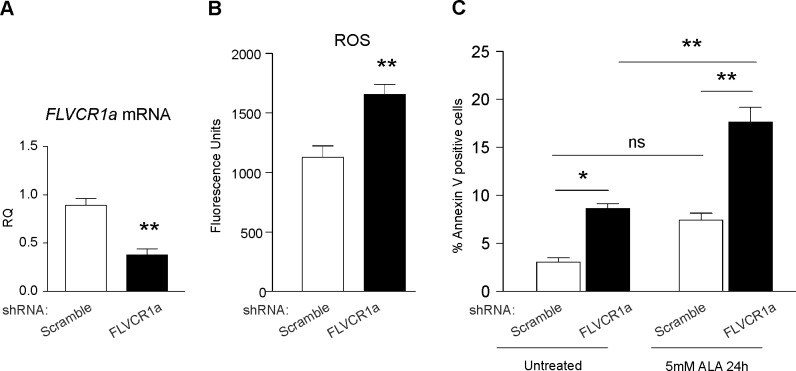
FLVCR1a is important for the survival of neuroblastoma cells. (**A**) qRT-PCR analysis of *FLVCR1a* mRNA levels in FLVCR1a-depleted SH-SY5Y cells compared to control (scramble). Values represent mean ± SEM. n = 3. ** = P<0.005. (**B**) Measurement of ROS levels mRNA in *FLVCR1a*-downregulated SH-SY5Y cells compared to controls. Values represent mean ± SEM. n = 6. ** = P<0.005. (**C**) Annexin V staining of *FLVCR1a*-downregulated SH-SY5Y cells compared to controls. Values represent mean ± SEM. n = 3. Two-way ANOVA. * = P<0.05; ** = P<0.005.

We previously reported that FLVCR1a is essential to prevent heme-induced oxidative stress in several cell types, including erythroid progenitors, hepatocytes and intestinal cells[21, 22, 33]. To get an insight into the role of FLVCR1a in neuronal cells, shRNA-mediated knockdown experiments were performed in SH-SY5Y neuroblastoma cells (Fig 4A and S2 Fig). Increased ROS levels were detected in *FLVCR1a*-downregulated SH-SY5Y cells compared to controls (Fig 4B), thus indicating a conserved role for FLVCR1a in neuroblastoma cells. Next, we investigated whether FLVCR1a is essential for the survival of these cells. Increased percentage of Annexin V-positive cells was detected in *FLVCR1a*-downregulated SH-SY5Y cells compared to controls, both under resting conditions and following the stimulation with ALA (Fig 4C). Together, these data indicate that heme export through FLVCR1a is important for the survival of neuronal cells.

## Discussion

Different mechanisms leading to sensory neurodegeneration and pain loss have been described. For example mutations in voltage-gated sodium channels, membrane-shaping proteins or the autophagy receptor FAM134B, which triggers turnover of the endoplasmic reticulum, are causative for sensory neuron loss[1, 5]. Altered sphingolipid metabolism, impaired neurotrophin signaling or epigenetic dysregulation likewise results in sensory neuron damage[34]. We here report patients with early-onset pain insensitivity because of mutations in the heme exporter *FLVCR1*. The finding suggests that heme metabolism is critically involved in sensory neuron maintenance.

The *FLVCR1* gene is under strong positive selection with Residual Variation Intolerance Score (RVIS) and Gene Damage Index (GDI) scores of 0.02 (55.45%) and 2854.439, respectively. Thus, the genic intolerance is estimated to be rather average and when testing for selective pressure, the GDI is classified as “medium damage prediction”. As part of the Major-Facilitator-Superfamily (MFS) FLVCR1 and other family members share 12 transmembrane alpha helices connected by hydrophilic loops. The subRVIS-prediction tool indicates that the crucial MFS domain of FLVCR1, in which all four mutations reported here are located, has a low RVIS score of -1.39 (3.96%), indicating a strong negative selection for this particular domain. This is compatible with pathogenicity of mutations residing in the conserved MFS domain. In contrast, the less well conserved N- and C-terminal regions of FLVCR1 show higher subRVIS scores of 0.07 (55.86%) and 1.12 (93.99%), respectively, which explains the overall higher RVIS score of FLVCR1.

FLVCR1 is a heme exporter involved in the control of intracellular heme levels in different cell types[20–25].

We demonstrated that sensory neuropathy-associated *FLVCR1*-mutations compromise the ability of FLVCR1a to export heme. Patient-derived primary cells showed altered expression of genes transcriptionally regulated by heme and accumulation of heme following the stimulation of endogenous heme synthesis. Thus, we propose that reduced heme export activity may cause heme accumulation and cellular damage by heme toxicity in sensory neurons.

The analysis of patient-derived cells also indicates that FLVCR1 impairment triggers compensatory mechanisms to counteract the accumulation of heme. Indeed, increased heme degradation was observed in patient fibroblasts whereas decreased heme synthesis rates were found in patient LCLs. However, these mechanisms fail to completely protect the cells from heme toxicity, especially under stress conditions, resulting in oxidative stress and increased susceptibility to programmed cell death. These data are in agreement with previous studies indicating an essential role for FLVCR1a to prevent heme-induced oxidative stress in different cell types[21, 22].

We here show a conserved role for FLVCR1a in neuronal cells. Silencing of FLVCR1a induces oxidative stress and affects the survival of neuroblastoma cells under resting conditions, thus suggesting that neuronal cells are particularly sensitive to the loss of FLVCR1a. We propose FLVCR1a as an important heme exporter in sensory neurons which regulates the oxidative stress response.

Mutations in the human *FLVCR1* gene were previously associated to Posterior Column Ataxia and Retinitis Pigmentosa (PCARP)[12]. PCARP is a childhood-onset, autosomal-recessive disorder with the clinical features of sensory ataxia and retinitis pigmentosa[11–14, 35]. Interestingly, polyneuropathy has been reported in a patient affected by PCARP, however, not as leading clinical manifestation but as additional symptom at a later state of disease[11]. In the majority of patients affected by PCARP, homozygous missense mutations in the FLVCR1 gene have been reported[12]. These mutations affect highly conserved residues of FLVCR1 important for protein expression, localization, folding or regulation of heme efflux[12]. Both patients reported here harbour a missense-mutation on one allele and a severe frameshift-mutation on the other *FLVCR1* alleles, the latter most likely resulting in a truncated FLVCR1a protein that may be rapidly degraded, acts as a dominant negative molecule or leads to nonsense-mediated mRNA decay (NMD). Such mutations are likely to cause severely impaired heme efflux rates, which may be particularly relevant for sensory neurons. This finding suggests that the type of mutation critically determines the phenotypic outcome of the disease.

It is well known that heme is involved in multiple biological processes[26], yet the role of heme metabolism in the nervous system has been poorly addressed. The finding of FLVCR1 mutations in patients with peripheral sensory neuropathy suggests that heme metabolism via FLVCR1a plays an essential role for maintenance of these specialized neurons. Heme is essential for ATP production through oxidative phosphorylation. Indeed, heme is the fundamental cofactor of cytochromes of the electron transport chain[36–38] and reduced heme synthesis cause a reduction of complex IV activity and ATP synthesis[39]. Peripheral nerves need efficient energetic metabolism because of a considerable length of the axon. For this reason, sensory neurons may be particularly sensitive to disturbances in heme homeostasis and dependent on heme synthesis for oxidative phosphorylation, to sustain both anterograde and retrograde trafficking along the extraordinarily long axonal processes. Interestingly, the nerve growth factor signaling cascade (NGF/TrkA) is essential for sensory neuron survival and mutations in both *NGF* and its receptor, TrkA, encoded by *NTRK1*, lead to sensory and autonomic neuropathy. Since heme is known to regulate NGF signaling[40], it is tempting to speculate that altered NGF signaling provides a pathophysiological link to the FLVCR1 disorder.

Heme levels are also directly involved in neuron excitability and nociception by modulating the activity of voltage-gated K+ channels. Voltage-gated K+ channels are a large family of K+ channels that open on membrane depolarization and contribute to maintain the resting potential and to determine the characteristics and frequency of action potentials[41]. Several studies highlight a pivotal role of K+ channels in pain processing[42, 43]. Knocking-down specific K+ channels subunits results in alteration of nociception in mouse models[43].

Heme directly binds to the large-conductance calcium-dependent Slo1 BK channels and Kv1.4 A-type K+ channels. The high affinity binding of heme to Slo1 decreases the frequency of channel opening leading to an inhibition of transmembrane K+ currents[44, 45]. Its binding to the N-terminal domain of Kv1.4 channels inhibits the fast inactivation of the channel, thus reducing cellular excitability[46].

Heme can also indirectly modulate the activity of K+ channels through the production of ROS and carbon monoxide. ROS modulation of K+ channel activity has already been reported[41] and CO plays multiple roles in nociception[47]. Moreover, it has been proposed that oxidative stress promotes the development of acquired forms of neuropathy, like diabetic neuropathy[48, 49] and chemotherapy-induced neuropathy[50, 51].

Taken together, our data emphasize a critical role of heme metabolism for homeostasis of sensory neurons and suggest FLVCR1 as a neuroprotective target.

## Materials and Methods

### Patients

Patients were recruited by the specialized outpatient services for Rare Diseases at the Gaslini Hospital in Genova (Patient 1) and of Clinical/Medical Genetics at the San Camillo-Forlanini Hospital in Rome (Patient 2), Italy. Both patients are of European origin, and from non-consanguineous families. Patient 1 samples were obtained from the “Cell Line and DNA Biobank from patients affected by Genetic Diseases” (Istituto Giannina Gaslini), member of Telethon Network of Genetic Biobanks (project no. GTB12001). Patient 1 underwent peripheral blood sampling for molecular testing, and skin punch biopsy for fibroblast culture and functional studies. Patient 2 underwent peripheral blood sampling for molecular testing and lymphocyte culture. Because of slow healing wounds the family dissented with skin biopsies. Both patients were evaluated by full clinical and detailed instrumental investigations, comprising neurophysiological assessment, brain and total spine MRI.

As controls, we used primary fibroblasts and LCLs derived from healthy donors. Control fibroblasts were obtained from the “Cell Line and DNA Biobank from patients affected by Genetic Diseases” (Istituto Giannina Gaslini), member of Telethon Network of Genetic Biobanks (project no. GTB12001). As control, we chose primary fibroblasts derived from an individual with comparable age to that of patient 1 (newborn child). Controls LCLs were derived from healthy donors at the Molecular Biotechnology Center, University of Torino, Italy. Control and patient samples derived from individuals belonging to the same genetic population (European origin).

The study was approved by the local ethics board of the University Hospital Jena (research ethics approval 3244-09/11) and of the San Camillo Forlanini Hospital Rome (research ethics approval OSR83/2016). Written consent for the study was obtained from the participants or their legally authorized representatives. This research is in accordance with the Helsinki Declaration.

#### Cell culture

Human neuroblastoma SH-SY5Y cell line (ATCC no. RL-2266) was propagated in DMEM/F12 medium (TermoFisher Scientific) supplemented with 10% heat-inactivated low-endotoxin fetal bovine serum (TermoFisher Scientific), 100 U/ml penicillin and 100 mg/ml streptomycin.

Control and patient-derived primary fibroblasts were propagated in RPMI 1640 medium (TermoFisher Scientific) supplemented with 15% heat-inactivated low-endotoxin fetal bovine serum (TermoFisher Scientific), 100 U/ml penicillin and 100 mg/ml streptomycin.

Control and patient-derived lymphoblastoid cell lines (LCLs) were propagated in RPMI 1640 medium (TermoFisher Scientific) supplemented with 20% heat-inactivated low-endotoxin fetal bovine serum (TermoFisher Scientific), 100 U/ml penicillin and 100 mg/ml streptomycin.

Cells were maintained at 37°C under a 5% CO2 atmosphere.

To stimulate the endogenous heme synthesis cells were treated with 5mM ALA (A3785; Sigma-Aldrich).

To facilitate heme efflux through FLVCR1a, LCLs were stimulated with 5μM or 15μM Hemopexin (CSL; Behring) under starved conditions.

#### FLVCR1a silencing

The downregulation of FLVCR1a in SH-SY5Y cells was achieved using a specific shRNA, as previously reported[18]. Briefly, a shRNA against the first exon of human FLVCR1 gene (Open Biosystem) was used to specifically down-regulate FLVCR1a expression. A shRNA against a scramble (SCR) sequence was used as control. Following lentiviral infection, cells were selected with 0.02μg/ml puromycin.

#### Whole-exome sequencing

For trio whole-exome sequencing, 50 ng of DNA from patients and parents were tagmented (Illumina). The fragments were adaptor-ligated including incorporation of sample index barcodes. Libraries were subjected to an enrichment process (Nextera Rapid Capture Exome Kit, Illumina). Samples were sequenced on a NextSeq550 platform. This resulted in 29 Gb of mapped sequences with a mean coverage of 250 and a 50x coverage of approx. 90% and a 10x coverage of 99.3% of target sequences per individual.

#### Gene panel sequencing

HaloPlex libraries for NGS were prepared using the HaloPlex Target Enrichment kit for custom design (1-500kb target region, Agilent Technologies) according to the manufacturer’s protocols. Target sequences (exons) and adjacent intronic (±50bp) sequences were enriched with the designed HaloPlex probes selected with Agilent’s SureDesign software. The panel included known disease-causative genes for hereditary pain disorders (genes implicated in HSAN1-HSAN8, or CIP) as well as functional candidate genes (genes encoding TRP-channels, ASICs, neurotrophins and their receptors). Libraries were run on a MiSeq Sequencer (Illumina) with a 300 cycle MiSeq reagent kit v2 (Illumina).

#### Processing of next-generation sequencing (NGS) data

Primary data were filtered accordingly to signal purity with the Illumina Real-Time Analysis (RTA) software v1.8. Reads were mapped against the human reference genome built 19 (hg19) using the Burrows-Wheeler-Aligner tool (bwa). Paired end reads were fixed by PICARD. Several steps to enhance data-quality were carried out using GATK v3.3, e.g. local realignment around short insertion and deletions and recalibration of the base quality scores. The GATK HaplotypeCaller was used to identify SNPs and INDELs. Subsequently, variant re-calibration was performed using stochastic models and divers training-sets (hapmap_3.3.hg19, 1000G_omni2.5.hg19, dbsnp_138.hg19,1000G_phase1.snps.high_confidence.hg19,Mills_and_1000G_gold_standard.indels.hg19). Detected variants were then annotated with the help of ANNOVAR. Specific filter-criteria were applied: focus on exonic and splicing regions; allele frequency of less than 0.01 in dbSNP, the 1000-Genomes project, the Exome Variant Server or the ExAC browser and in an in-house database; and prioritization for *de novo* mutations and compound-heterozygous / homozygous variants.

#### Sanger sequencing

Sanger sequencing was performed using standard procedures. Automated electrophoreses was done on a capilary electrophoretic ABI 3130 genetic analyzer (Applied Biosystems).

#### Immunoprecipitation and western blotting

Control and patient-derived fibroblasts and LCLs were lysed in TBS1X;Triton1% supplemented with protease inhibitors (Roche). Cell debris was removed by centrifugation at 13,000 g for 10 min and the supernatant was collected. 50μg of total protein extracts were used for western blotting. For immunoprecipitation, 1mg of total protein extracts were immunoprecipitated with anti-FLVCR1 antibody (Abnova H00028982-D01P). Anti-SUMO (Thermo Fisher Scientific 519100) antibody was used as control.

Proteins were separated on 10% SDS–PAGE, transferred on nitrocellulose membrane and incubated with antibodies specific for FLVCR1 (Abnova H00028982-D01P), HO1 (ENZO ADI-SPA-896F), ALAS1 (Abcam ab84962) and β-actin (Sigma). Detection of immunoblots was carried out with ECL (Biorad).

#### RNA extraction and quantitative real-time PCR analysis

Total RNA was extracted using PureLink RNA Mini Kit (TermoFisher Scientific). For quantitative real-time PCR (qRT-PCR), 1 μg total RNA was treated with DNAse (Promega) and transcribed into complementary DNA (cDNA) using High-Capacity cDNA Reverse Transcription Kit (TermoFisher Scientific). qRT-PCR was performed on a 7300 Real Time PCR System (Applied Biosystems) using Universal Probe Library System (Roche). See S3 and S4 Tables for details.

#### Measurement of heme content

Intracellular heme concentration of control and patient-derived cells, as well as SH-SY5Y cells, was measured using a fluorescence assay, as previously reported[52]. Briefly, cells were collected and resuspended in 2 M oxalic acid and heated at 95°C for 30 minutes, leading to iron removal from heme. The resultant protoporphyrin was measured by fluorescence (400 nm excitation and 662 or 608 nm emission). Data were normalized to the endogenous protoporphyrin content (by measuring the fluorescence of not-heated samples) and to total protein concentration. Results were expressed as pmol of heme/mg total protein

#### Measurement of intracellular ROS accumulation

Accumulation of reactive oxygen species (ROS) in control and patient-derived cells, as well as SH-SY5Y cells, was assessed by using the oxidant-sensitive fluorescent dye 29,79-dichlorodihydrofluoroscein diacetate (H_2_DCFDA; Molecular Probes, Inc., Eugene, OR)[21, 33]. H2DCFDA penetrates easily into the cells. Upon crossing the cellular membrane, H2DCFDA undergoes deacetylation by intracellular esterases producing a nonfluorescent compound that becomes highly green fluorescent following oxidation by intracellular reactive oxygen species. Within the cell, the probe reacts with ROS to form fluorescent 28,78 dichlorofluoroscein (DCF), which is detected by fluorometry. Control and patient-derived cells, as well as SH-SY5Y cells, were incubated with 5mM H2DCFDA in Hanks’ balanced salt solution (HBSS) for 30 min at 37°C under 5% CO2 atmosphere. atmosphere. Then, cells were washed twice with 0.1 M PBS and lysed in 0.1 M PBS. A quantity of lysate correspondent to 10 μg protein was analyzed. Fluorescence was recorded at excitation and emission wavelengths of 485 and 530 respectively by a fluorimeter plate reader (Promega). The background fluorescence caused by buffer and DCF was subtracted from the total fluorescence in each well generated by cells in presence of DCF.

### Annexin V staining

Control and patient-derived cells, as well as SH-SY5Y cells, were collected, washed in PBS1X, resuspended in 10 mM Hepes, 150 mM NaCl, 5 mM CaCl_2_ buffer, and labelled with AnnexinV-FITC (BD Biosciences) for 15 minutes. Then, 2 μl propidium iodide (1 mg/ml) (PI; Sigma-Aldrich) was added. AnnexinV emission was detected in the green channel (525 nm) and propidium iodide in the red channel (575nm) on a FACS Calibur Cytometer (BD Biosciences). For each sample, 10’000 to 25’000 events were collected.

### Statistical analyses

Results were expressed as mean ± SEM. Statistical analyses were performed using one-way or two-way analysis of variance or Student’s *t* test. A *P* value of less than 0.05 was considered significant.

## Supporting Information

S1 Fig (**A**) Mild hyperintensity of the posterior columns at the cervical metameteres in patient 2. (**B**) cervical syringomyelia in the same patient.
(TIF)

S2 Fig (**A**) qRT-PCR analysis of *FLVCR1b* mRNA levels in patient 1 and control fibroblasts. Values represent mean ± SEM. N = 6. (**B**) qRT-PCR analysis of *FLVCR1b* mRNA levels in patient 2 and control LCLs. Values represent mean *FLVCR1b* mRNA levels compared to the mean *FLVCR1b* mRNA levels of 4 different control LCLs. (**C**) qRT-PCR analysis of *FLVCR1b* mRNA levels in *FLVCR1a*-downregulated SH-SY5Y cells compared to controls (scramble). Values represent mean ± SEM. N = 6.
(TIF)

S3 Fig Densitometric analysis of HO1 and ALAS1 protein levels in patient 2 LCLs compared to the mean protein levels of 4 different control LCLs. (TIF)

S4 Fig Annexin V-positive cells in patient 2 compared to control LCLs, under basal conditions and following the stimulation with 5μM H_2_O_2_ for 72 hours. Values represent mean ± SEM. n = 3. ** = P<0.005; *** = P<0.001.
(TIF)

S5 Fig qRT-PCR analysis of *FLVCR1a* mRNA levels in different wild-type mouse tissues. Values represent mean ± SEM. n = 5. ** = P<0.005; *** = P<0.001.
(TIF)

S1 Table Whole-exome sequencing data. *De novo* variants as well as compound heterozygous and homozygous variants are given for both trios.
(XLSX)

S2 Table In silico prediction. Pathogenicity of FLVCR1 variants is predicted by several prediction programs.
(XLSX)

S3 Table Primers and probes used for qRT-PCR analyses. The primers and probes were designed using the ProbeFinder Software (Roche). Human β-actin (TermoFisher Scientific) was used as endogenous control.
(PDF)

S4 Table Primers and probes used for qRT-PCR analyses. To discriminate between FLVCR1a and FLVCR1b, specific primers and probes were designed using Primer Express Software Version 3.0 (Applied Biosystems). Human β-actin (TermoFisher Scientific) was used as endogenous control.
(PDF)
