# Supplementary figures and images for: Mutations in the Heme Exporter FLVCR1 Cause Sensory Neurodegeneration with Loss of Pain Perception
Source: PLoS Genet. 2016 Dec 6;12(12):e1006461. doi: 10.1371/journal.pgen.1006461 (PMC5140052; doi:10.1371/journal.pgen.1006461)

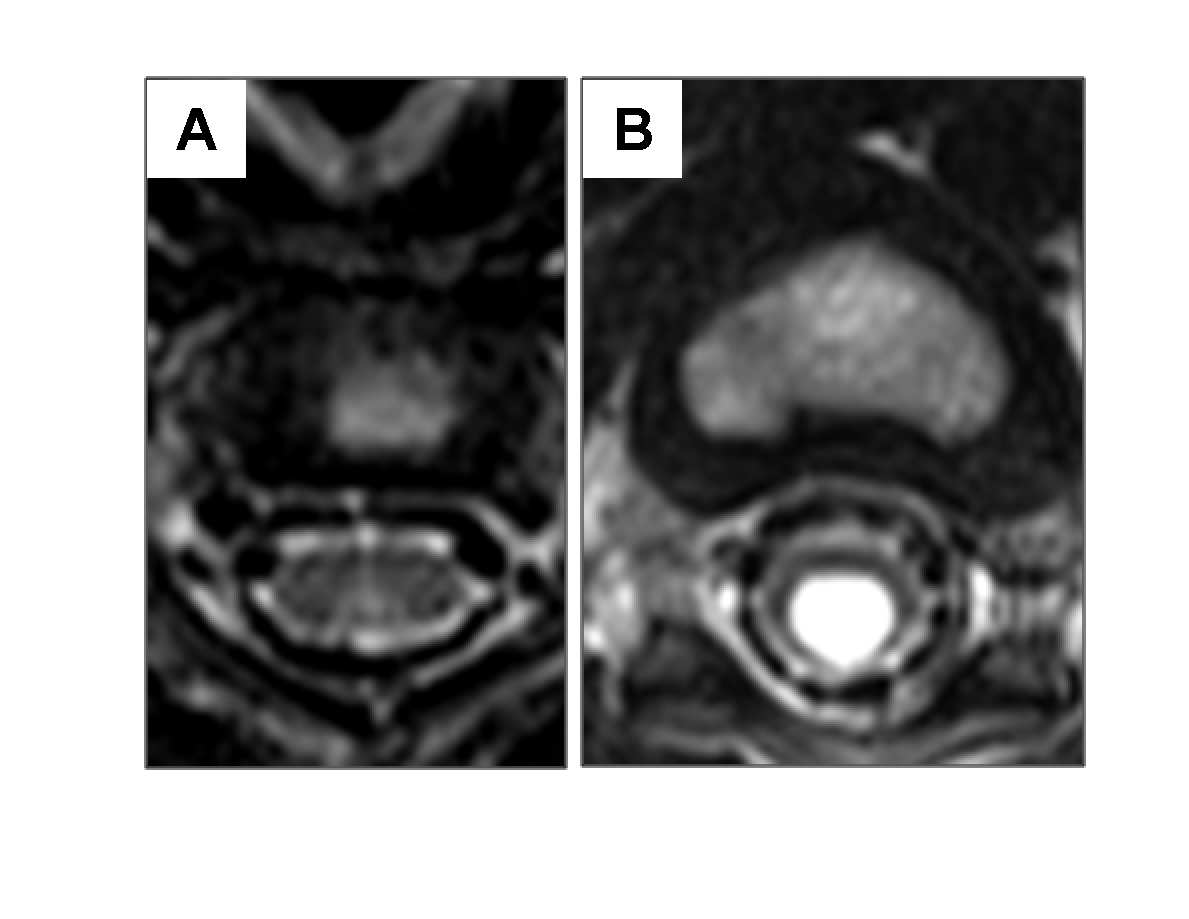

Supplement: S1 Fig — (A) Mild hyperintensity of the posterior columns at the cervical metameteres in patient 2. (B) cervical syringomyelia in the same patient. (TIF) [file pgen.1006461.s001.tif]

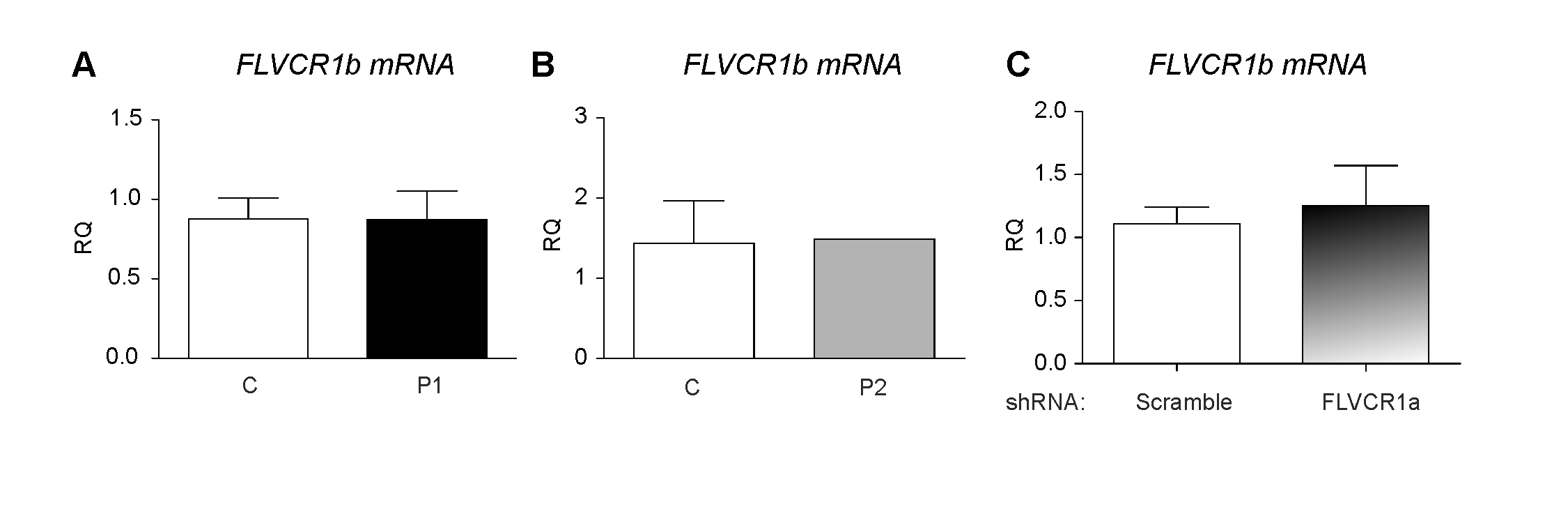

Supplement: S2 Fig — (A) qRT-PCR analysis of FLVCR1b mRNA levels in patient 1 and control fibroblasts. Values represent mean ± SEM. N = 6. (B) qRT-PCR analysis of FLVCR1b mRNA levels in patient 2 and control LCLs. Values represent mean FLVCR1b mRNA levels compared to the mean FLVCR1b mRNA levels of 4 different control LCLs. (C) qRT-PCR analysis of FLVCR1b mRNA levels in FLVCR1a-downregulated SH-SY5Y cells compared to controls (scramble). Values represent mean ± SEM. N = 6. (TIF) [file pgen.1006461.s002.tif]

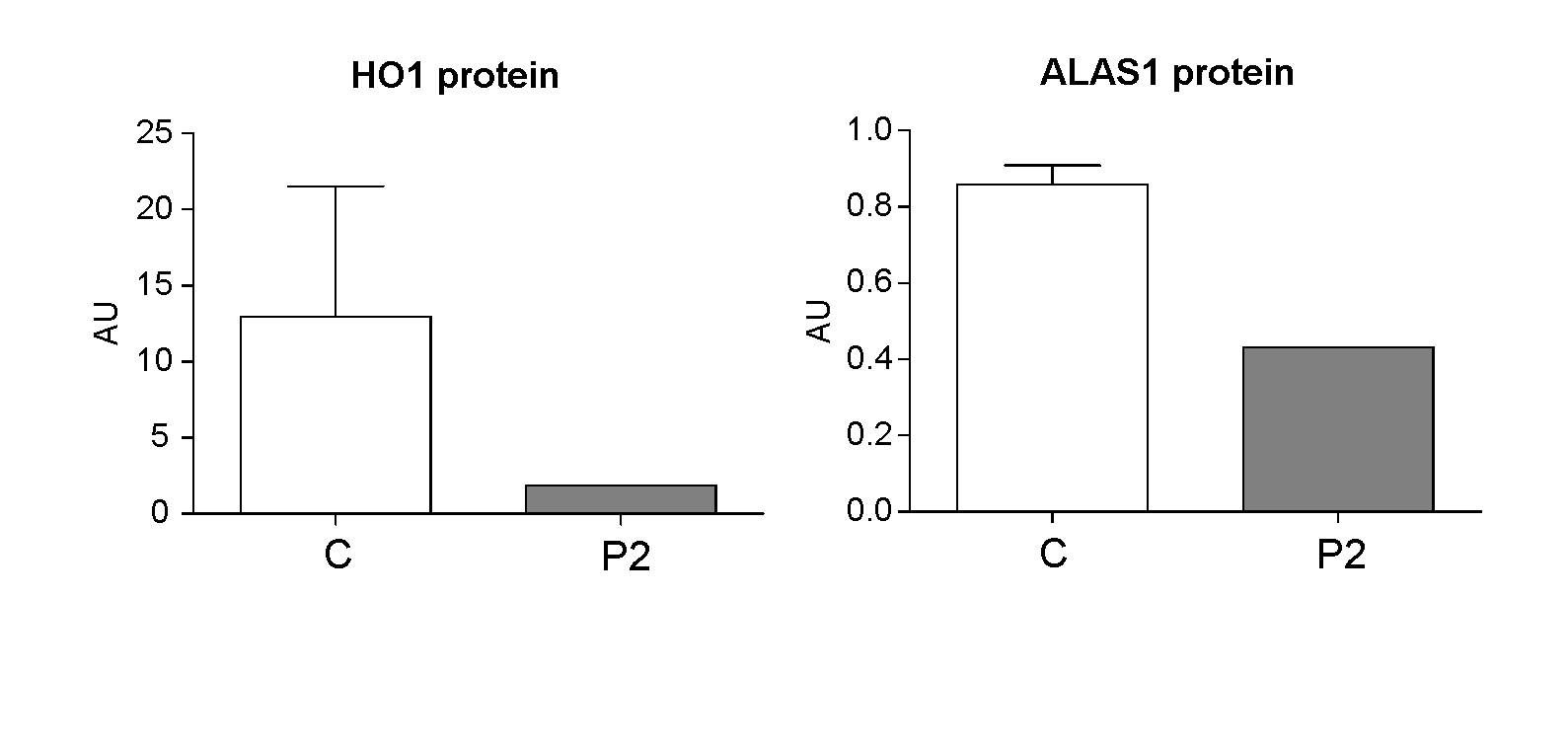

Supplement: S3 Fig — (TIF) [file pgen.1006461.s003.tif]

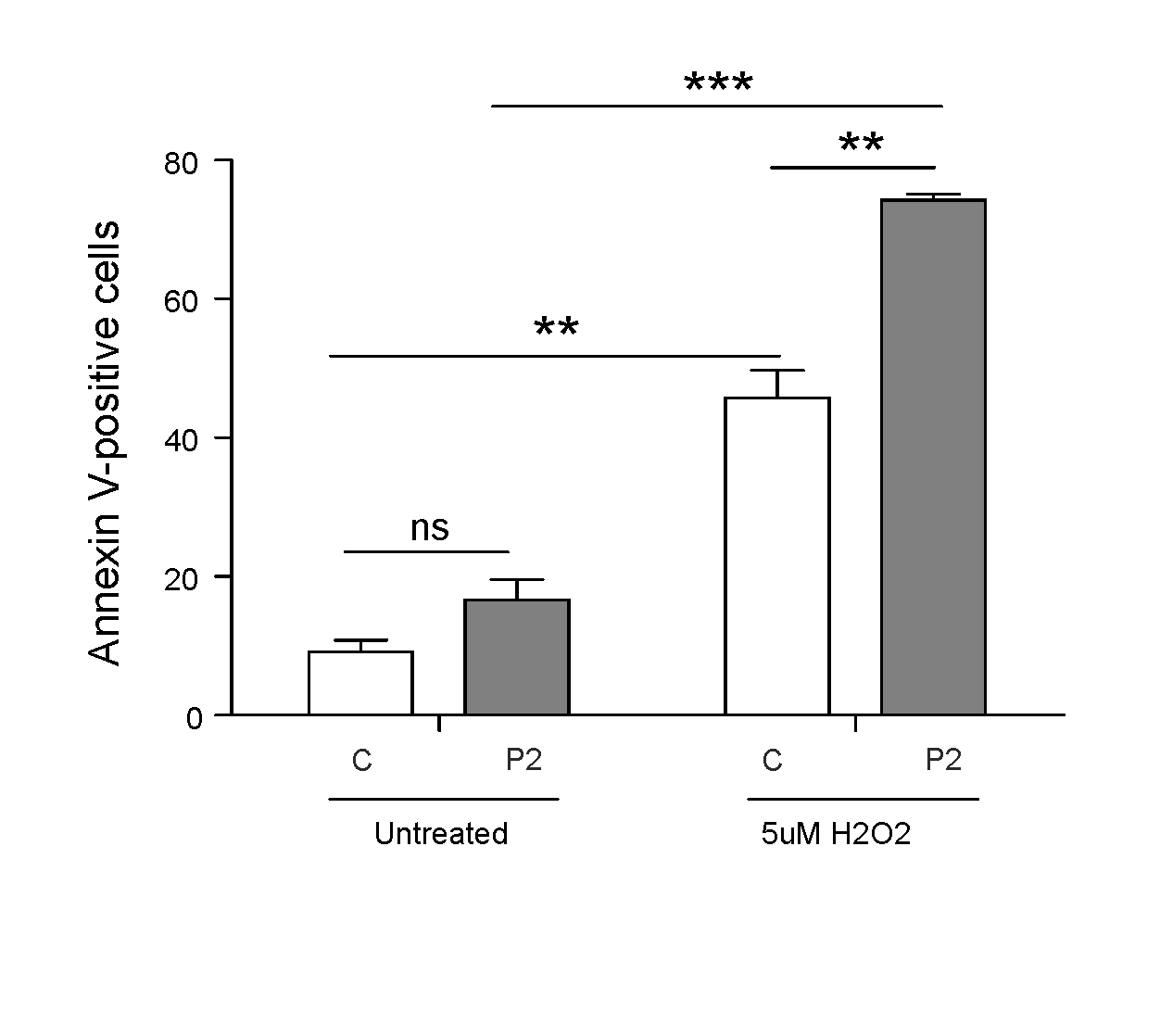

Supplement: S4 Fig — Values represent mean ± SEM. n = 3. ** = P<0.005; *** = P<0.001. (TIF) [file pgen.1006461.s004.tif]

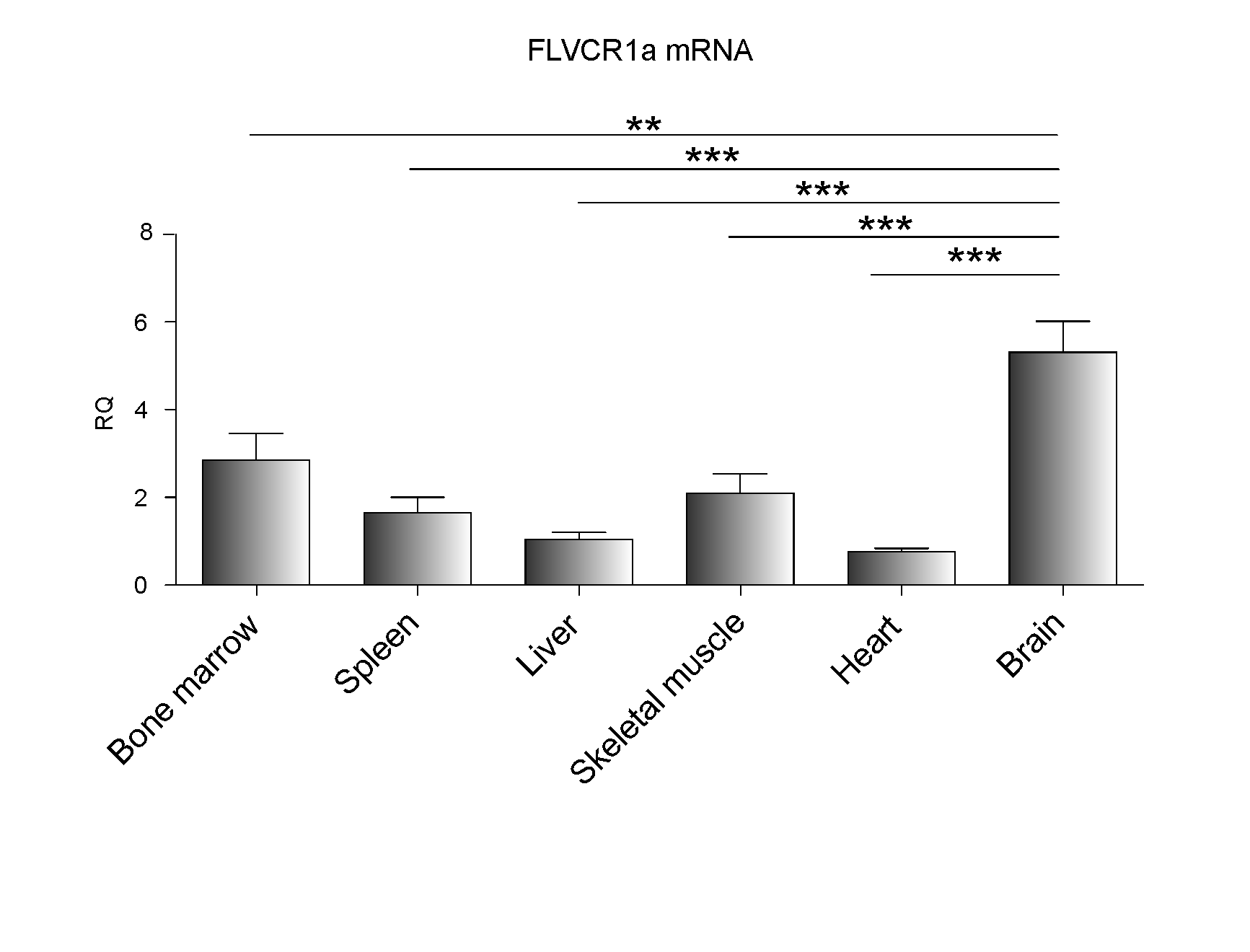

Supplement: S5 Fig — Values represent mean ± SEM. n = 5. ** = P<0.005; *** = P<0.001. (TIF) [file pgen.1006461.s005.tif]
