# Supplementary material for: Mutations in the Heme Exporter FLVCR1 Cause Sensory Neurodegeneration with Loss of Pain Perception
Source: PLoS Genet. 2016 Dec 6;12(12):e1006461. doi: 10.1371/journal.pgen.1006461 (PMC5140052; doi:10.1371/journal.pgen.1006461)
Supplement: S3 Table — The primers and probes were designed using the ProbeFinder Software (Roche). Human β-actin (TermoFisher Scientific) was used as endogenous control. (PDF) [file pgen.1006461.s008.pdf]

Gene Name	Forward Primer	Reverse Primer	Probe (Universal Probe Library-Roche)
ALAS1	GAAATGAATGCCGTGAGGAA	CCTCCATCGGTTTTCACACT	#40
HO1	GGGTGATAGAAGAGGCCAAGA	AGCTCCTGCAACTCCTCAAA	#42
FPN	GCTCTAGCTGTGAAAGCTGGTC	AGTTCCCTCCAGGGGTTTT	#35
FT-H	GCCAGAACTACCACCAGGAC	CATCATCGCGGTCAAAGTAG	#1
FT-L	GCTGAACCAGGCCCTTTT	TCCAGGAAGTCACAGAGATGG	#37
DMT1	CATGCTGGCCTCTTTCCTAA	AACATCCCAGCACAACACC	#76
SOD1	TCATCAATTTCGAGCAGAAGG	GCAGGCCTTCAGTCAGTCC	#60
SOD2	AATCAGGATCCACTGCAAGG	TAAGCGTGCTCCCACACAT	#3
CATALASE	TCATCAGGGATCCCATATTGTT	CCTTCAGATGTGTCTGAGGATTT	#76
THIOREDOXIN1	TGAGGAGAAAGCTGTGGAGAA	CCATTCCAATGGCCAAAA	#60

Table S3
