# Supplementary material for: Mutations in the Heme Exporter FLVCR1 Cause Sensory Neurodegeneration with Loss of Pain Perception
Source: PLoS Genet. 2016 Dec 6;12(12):e1006461. doi: 10.1371/journal.pgen.1006461 (PMC5140052; doi:10.1371/journal.pgen.1006461)
Supplement: S4 Table — To discriminate between FLVCR1a and FLVCR1b, specific primers and probes were designed using Primer Express Software Version 3.0 (Applied Biosystems). Human β-actin (TermoFisher Scientific) was used as endogenous control. (PDF) [file pgen.1006461.s009.pdf]

Gene	Forward Primer	Reverse Primer	Probe
FLVCR1a	TTGGGCCCAAAGAGGTGTC	GCCAGGAGATTTGTGTCATTCTG	6FAM-ACCACCAGTTTTAGTACCCAA-MGB
FLVCR1b	TCCTCTTTATGTTCTGTTAATTGCCA	GCCAGGAGATTTGTGTCATTCTG	6FAM-ACCACCAGTTTTAGTACCCAA-MGB

Table S4
